# Medical school origins of award-winning anaesthetists; analysis of a complete national dataset

**DOI:** 10.1186/s12909-024-05873-6

**Published:** 2024-08-19

**Authors:** Sinclair Steele, Abdulaziz Shalaby, Mustafa Khafaja, Gabriel Andrade

**Affiliations:** College of Medicine, Ajman University, University Street, Al Jerf 1, Ajman, United Arab Emirates

**Keywords:** Anaesthetists, Medical schools, Medical careers, Award-winners, Globalization, International Medical Graduates

## Abstract

**Background:**

The ultimate aim of medical education is to produce successful practitioners, which is a goal that educators, students and stakeholders support. These groups consider success to comprise optimum patient care with consequently positive career progression. Accordingly, identification of the common educational features of such high-achieving doctors will facilitate the generation of clinical excellence amongst future medical trainees. In our study we source data from British clinical merit award schemes and subsequently identify the medical school origins of anaesthetists who have achieved at least national distinction.

**Methods:**

Britain operates Distinction Award/Clinical Excellence Award schemes which honour National Health Service doctors in Scotland, Wales and England who are identified as high achievers. This quantitative observational study used these awards as an outcome measure in an analysis of the 2019–20 dataset of all 901 national award-winning doctors. Where appropriate, Pearson's Chi-Square test was applied.

**Results:**

The top five medical schools (London university medical schools, Edinburgh, Dundee, Aberdeen and Glasgow) were responsible for 56.4% of the anaesthetist award-winners, despite the dataset representing 85 medical schools. 93.6% of the anaesthetist merit award-winners were from European medical schools. 8.06% of the anaesthetist award-winners were international medical graduates compared with 11.5% non-anaesthetist award-winners being international medical graduates.

**Conclusions:**

The majority of anaesthetists who were national merit award-winners originated from only five, apparently overrepresented, UK university medical schools. In contrast, there was a greater diversity of medical school origins among the lower grade national award-winners; tier 3 award-winners represented 20 different medical schools from three continents. As well as ranking educationally successful university medical schools, this study assists UK and international students, by providing a roadmap for rational decision making when selecting anaesthetist and non-anaesthetist medical education pathways that are more likely to fulfil their career ambitions.

## Background

Historically, In Britain there have been two clinical merit award schemes established to reward successful clinicians employed in the National Health Service (NHS):

(i) The Clinical Excellence Awards (CEA) scheme, Covering Wales and England [1].
(ii) The Distinction Awards (DA) scheme, Covering Scotland [1]

The schemes are similar in aims and organization; both offer tiers of local and national awards to high-achieving doctors. However, the CEA scheme is currently being restructured, renamed and re-established as the National Clinical Impact Awards (NCIA), whilst the DA scheme remains in place in Scotland. The doctors receiving such awards gain benefits not only from the effects of these honours on their reputations and career progressions but also from the recurring financial rewards accompanying such accolades [1].

These UK national award schemes were envisioned and implemented after World War II for the pragmatic purpose of motivating senior NHS. Since their inception, these schemes and their implementation clinicians to support the newly-created have been the cause of vigorous debate in the UK medical community. As a result, these clinical merit awards have been evaluated and discussed with regard to bias from the perspective of award objectivity [2], specialty distribution [3], regional distribution [3], gender parity [1], age distribution [4] and ethnicity/racial distribution [5] but, until our research series, *not by medical school of origin*. These constructive criticisms have resulted in iterative revisions of these award schemes over the recent three decades. Many medical commentators agree that there should be a system to reward high-achieving clinicians [6] and the CEA/DA/NCIA merit awards are seen as national recognition of clinical career success—accounting for their continuing value, greater than 70 years after their inception. This original innovative research study is part of a series that contributes to the medical education discussion by relating the anaesthetist and non-anaesthetist merit award-winners to their *medical schools of origin*. We place our findings in the contexts of educational, career and global implications for ambitious prospective medical students, undergraduate medical students and doctors aspiring to attain career success [7, 8].

## Methods

The lists of anaesthetist award-winners and non-anaesthetist award-winners were retrieved from the source material of the DA annual report (Scotland) for 2019–2020 [9] together with the CEA annual report (England and Wales) [10] for the 2019–2020 awards round. These lists were summations of both the newly selected awardees and the previous award-winners who had retained their awards. The medical schools of origin were identified by using the published Medical Register, UK [11] and the published Dental Register, UK [12–14].The total number of award-winners was 901; the university medical schools of origin were successfully identified for 99.8% of these clinicians [13, 14]. Accordingly, 899 doctors were included in the analyzed dataset. Award-winning doctors in the publications above, who were designated as specializing in the anaesthetic discipline, were included in this study following the methods protocol established by our earlier articles in this series [13, 14].

The rankings of medical schools by number of merit award-winning alumni were determined by summation of the number of anaesthetist award-winners of A plus (A^+^), A or B grade (or platinum, gold, silver or bronze award-winners) [13, 14]. Only these national level Clinical Excellence Awards and Distinction Awards were included in this study [13, 14]. Combining these parallel and similar award gradings, permitted all of Britain's (England, Wales and Scotland) excellence award-winners to be analyzed together [13, 14]. As part of our analysis of the grades of awards we collated the award categories to explicitly show the three tiers of national merit awards; A plus and platinum award-winners were combined to yield the top tier (tier 1) of national anaesthetist awards [13, 14]. The A and gold awards were combined to create the intermediate tier (tier 2) of national anaesthetist awards [13, 14]. Finally, the B and silver/bronze awards were combined to create the lowest tier (tier 3) of national anaesthetist merit awards [13, 14]. The same approach was taken with the non-anaesthetist data [13, 14].

The rankings of the medical schools by the number of merit award-winning alumni were approximately size corrected by dividing the total number of award-winners that were alumni of the medical school by the number of admissions to the undergraduate medical school in the 2019–20 academic year [13, 14]. We used this pragmatic approach to estimate the size correction rather than the more ideal but inaccessible integral of medical school graduation numbers against time for approximately the last 50 years [13, 14]. The comparison of the distributions of award-winners (anaesthetist merit award-winners versus non-anaesthetist merit award-winners) was quantified using Pearson's Chi-Square test with the significance level set to *p* < 0.05 [13, 14].

On the basis of the frequency of award holders in the 2019–20 round, the top 20 medical schools were selected. For those 20 medical schools, a Pearson’s coefficient was calculated to determine the correlation between the age of the medical school by establishment date and the number of award-winners corrected by size (award- winners/number of admissions 2019–20).

All procedures were performed in compliance with the pertinent guidelines [13, 14]. Patients and public involvement; no patient involvement [13, 14]. The methods that were applied in our study, and that cover the description in this methods section, were similar to and closely derived from earlier publications in this series, which we cite here [13, 14].

## Results

There were 62 anaesthetist award-winners in the 2019–20 award round.

Table 1 shows the ten medical schools that attained the largest number of alumni merit award-winners; these award-winners possessed platinum, gold, silver, bronze, A plus, A or B awards. More importantly, Table 1 compares the originating medical schools of the anaesthetist and non-anaesthetist merit award-winners for the ten medical schools with the largest numbers of award-winners; the table contrasts the numbers and percentages of anaesthetist award-winners and non-anaesthetist award-winners which the graduates of each medical school attained. Pearson's Chi-Square test demonstrated that there was not a statistically significant difference between the distributions of the medical schools of origin for anaesthetist merit award-winners versus the non-anaesthetist merit award-winners, *p* > 0.05. Graduates of London university medical schools, Edinburgh, Dundee, Aberdeen and Glasgow medical schools accounted for 56.4% of anaesthetist award-winners. In comparison, 58.4% of the non-anaesthetist merit award-winners were graduates of six British medical schools: London university medical schools, Glasgow, Edinburgh, Aberdeen, Cambridge and Oxford.

**Table 1:** Top 10 medical schools; analysis by number of anaesthetist award holders, number of non-anaesthetist award holders and total number of award holders

Medical school	Total number of award holders	Number of anaesthetist award holders	Percentage of anaesthetist award holders	Number of non-anaesthetist award holders	Percentage of non-anaesthetist award holders
London	179	12	19.4	167	20.0
Glasgow	113	4	6.45	109	13.0
Edinburgh	84	11	17.7	73	8.72
Aberdeen	60	4	6.45	56	6.69
Oxford	45	3	4.84	42	5.02
Cambridge	43	1	1.61	42	5.02
Manchester	38	3	4.84	35	4.18
Birmingham	29	2	3.23	27	3.23
Dundee	29	4	6.45	25	2.99
Nottingham	26	2	3.23	24	2.87

Table 2 displays the effect of the approximate medical school size correction on the ranking of the medical schools by number of alumni award-winners. London's number one ranking (anaesthetists) before size correction dropped to a number seven ranking after size correction. Similarly, Dundee's number nine ranking (non-anaesthetists) before size correction became a number five ranking after size correction.

**Table 2:** Top 10 medical school rankings by number of graduates holding merit awards; with or without size correction

Medical school	Total number of anaesthetist award holders	Ranking by number of anaesthetist award holders	Ranking by anaesthetist award holders after size correction	Total number of non-anaesthetist award holders	Ranking by number of non-anaesthetist award holders	Ranking by non-anaesthetist award holders after size correction
Glasgow	4	3 =	4	109	2	1
Edinburgh	11	2	1	73	3	2
Oxford	3	6 =	3	42	5	3
Aberdeen	4	3 =	5	56	4	4
Dundee	4	3 =	2	25	9	5
Cambridge	1	10	10	42	6	6
London	12	1	7	167	1	7
Manchester	3	6 =	6	35	7	8
Birmingham	2	8 =	9	27	8	9
Nottingham	2	8 =	8	24	10	10

Our analysis included a comparison of the anaesthetist A plus/platinum award-winners (designated tier 1) with A/gold award-winners (designated tier 2) and B/silver/bronze award-winners (designated tier 3). The tier 1 anaesthetist award-winners originated from 3 medical schools: Madras, London university medical schools and Newcastle. The tier 2 anaesthetist award-winners came from 9 medical schools: Aberdeen, Birmingham, Bristol, Dundee, Edinburgh, Glasgow, London university medical schools, Newcastle and Oxford. The tier 3 anaesthetist award-winners originated from 20 medical schools: Aberdeen, Amravati, Basra, Birmingham, Bristol, Cambridge, Cape town, Dublin, Dundee, Edinburgh, Glasgow, Liverpool, London university medical schools, Malta, Manchester, Newcastle, Nottingham, Oxford, Sheffield and Southampton.

Table 3 contrasts the continental locations of the originating medical schools for anaesthetist and non-anaesthetist merit award-winners, for the ten medical schools with the greatest numbers of award-winners. 93.6% of anaesthetist merit award-winners were from European medical schools, in comparison 91.5% of the non-anaesthetist award-winners were from European medical schools. Pearson's Chi-Square test indicated that there was not a statistically significant difference between the continental locations of the originating medical schools for anaesthetist and non-anaesthetist merit award-winners, *p* < 0.05.

**Table 3:** A geographical comparison of the medical schools of origin of anaesthetist and non-anaesthetist merit award holders

Continental location of medical school	Non-Anaesthetists		Anaesthetists	
	Total number of non-anaesthetist award holders	Percentage of total number of non-anaesthetist award holders	Total number of anaesthetist award holders	Percentage of total number of anaesthetist award holders
Africa	18	2.15	1	1.61
Asia	38	4.54	3	4.84
Australasia	9	1.08	0	0.00
Europe	766	91.5	58	93.6
North America	5	0.60	0	0.00
South America	1	0.12	0	0.00
Total	837	100.0	62	100.0

8.06% of the anaesthetist award-winners were IMGs—in contrast, 11.5% of the non-anaesthetist award-winners were IMGs. The anaesthetist tier 1 award-winners included the greatest proportion of IMG award-winners at 33.3%.

After evaluating the top 20 university medical schools (arranged on the basis of award-winners’ frequencies), a moderate and positive correlation was found between the age of the medical school by establishment date and the number of award-winners corrected by size (number of admissions), *r*(18) = 0.47, *p* = 0.04.

Table 4 quantifies the number of award-winners per specialty for the eight specialties that accumulated the greatest number of award-winners.

**Table 4:** Comparison of number of national merit-awards per clinical specialty

Specialty-Group Rankings	Number of award-winners	Percentage of award-winners
1. Medical disciplines	337	37.5%
2. Surgical disciplines	224	24.9%
3. Laboratory medicine disciplines	95	10.6%
4. Anaesthetics	62	6.90%
5. Paediatric disciplines	48	5.33%
6. Psychiatric disciplines	44	4.89%
7. Public health medicine	34	3.78%
8. Radiological disciplines	32	3.56%

## Discussion

### Anaesthetist merit awards and UK medical schools

Our study is part of the first series to comprehensively analyze British clinical merit award-winners' medical schools of origin. This project identifies medical schools that have facilitated the successful medical education of anaesthetists by using the outcome measure of clinical merit award-winning. As a result, the data and analysis we provide will be of significance to local potential medical students as well as current and future graduates of International Medical Programs [15]. Our series of studies are the first to *rank medical schools by the number of merit award-winners* originating from each school, and accordingly will provide a new comparative perspective for medical educators.

The UK has been well known to attract international medical graduates to practise medicine. This was further confirmed and quantified in the General Medical Council 2019 workforce study that stated "For the first time, more non-UK medical graduates took up a licence to practise than UK medical graduates [16]." As a result of such workforce migrations, the scope of possible medical schools of origin of merit award-winners has essentially become global. Specifically, our database of merit award-winners covering the 2019–20 round has 85 different medical schools represented. This study shows that after being chosen by a "transparent and defensible" assessing and scoring arrangement [17] 56.4% of the anaesthetic award-winners received their undergraduate training at one of only five UK medical schools (Table 1). These were London university medical schools, Edinburgh, Dundee, Aberdeen and Glasgow. A similar pattern of concentration occurred amongst the non-anaesthetist merit award-winners; 58.4% of these were graduates of London university medical schools, Glasgow, Edinburgh, Aberdeen, Cambridge or Oxford. The observation that there is a similar concentration of award-winners amongst graduates of similar medical schools, for both the anaesthetists and non-anaesthetists, implies that there may be common underlying non-specialty specific factors which account for the success of these doctors. The quality of undergraduate medical education may well be such a factor.

Considering the data presented in Table 2 (where a size correction is applied) the top five medical schools of origin of the anaesthetists include Oxford and Edinburgh, so in this instance the prestige and good quality of medical education would seem to coincide in these universities [18]. Based on our data, a strong local or international student candidate applying to medical school who has a desire to specialize as a anaesthetist could be advised to favour Oxford, Edinburgh and Dundee medical schools, whereas a less strong student applicant who definitely did not want to specialize as an anaesthetist might be wiser to prioritize Glasgow or Aberdeen. A student who was not sure whether they preferred an anaesthetic or non-anaesthetic career pathway could consider Oxford and Edinburgh medical schools. Thus, the rankings of medical schools that we have produced in this study provide data which future prospective medical students can use to aid the selection of medical schools appropriate for their ambitions; this being particularly true for graduate students and mature students who often have strong career preferences even at the beginning of training. Students generally make rational decisions in the field of education [19, 20] and ranking information of this type is particularly important to an educational pathway as complex and tortuous as attempting to train to be a doctor in a particular specialty. Recent studies have demonstrated that the differences between medical schools tend to remain stable over time [21], so the guidance offered here will have valuable longevity. We understand that this type of ranking information is more likely to be of value to the graduate entry, mature students and international medical graduates than local undergraduate students who are unlikely to decide on their career specialty until the end of their medical degree.

Our observation of the concentration of award-winning anaesthetists and non-anaesthetists amongst a small number of medical schools prompted a consideration of the role of medical school size on the rankings. Specifically, after summation of the number of yearly graduates, London medical schools combine to be one of the largest medical schools in Europe. Therefore, as a proportion, London university medical schools' graduates would probably be well represented in any essentially Eurocentric merit award schemes. To investigate this, we performed an approximate size correction to the medical school rankings by number of award-winners, as described and discussed in the methods section, using the 2019 medical school student admission numbers. Applying this to the anaesthetist award-winners rankings, London university medical schools dropped from a position of one before the approximate size correction to a position of seven after size correction. A parallel effect occurred when the approximate size correction was applied to the non-anaesthetist award-winning rankings; here London university medical schools dropped in ranking from one to seven. Clearly, medical school size affects the medical school ranking. However, it is improbable that size alone can account for the concentration of clinical merit award-winners in a few medical schools; a factor related to the quality of the undergraduate medical education is also consistent with our findings.

### Anaesthetist merit awards and international medical schools

In view of the trend of medical trainees and students to travel internationally in this age of globalization [22, 23] we evaluated the originating medical schools of the award-winners by continent of university medical school origin. Table 3 shows a comparison of the continental origin of medical schools for anaesthetist and non-anaesthetist merit award-winners. 93.6% of the anaesthetist award-winners originated from European medical schools whereas 91.5% of the non-anaesthetist award-winners were originally trained in European medical schools. In statistical terms, there was not a significant difference between the continental locations of the originating medical schools, anaesthetists vs non-anaesthetists, p > 0.05 (Chi-square). We also noted that despite the apparent similarities between US and UK medical cultures, there were no anaesthetist merit award-winners who originated from USA medical schools. The fact that a similar statement could be made for non-anaesthetist award-winners implies that specialty specific factors are not responsible for this phenomenon.

Our research demonstrates a larger diversity of medical school origins among the lowest tier of anaesthetist award-winners than the highest tier of anaesthetist award-winners. More precisely, the anaesthetists with tier 1 awards came from 3 medical schools representing 2 continents, whereas the tier 3 anaesthetist award-winners originated from 20 medical schools representing 3 continents. These results seem to indicate a trend towards greater globalization and inclusivity in the lowest tier national merit awards. We believe future longitudinal analyses of merit award-winners would be important in accurately determining whether the tendency towards diversity extends to the higher tier and more prestigious awards.

Only 8.06% of the anaesthetist merit awards were held by IMGs whereas 11.5% of the of the non-anaesthetist merit awards were held by IMGs. Despite 16% of all hospital doctors being anaesthetists [24] our series shows that anaesthetists only hold 6.9% of all the merit awards (Table 4). The medical educational implications of these observations include that:

- 1) There will be fewer high profile "successful" anaesthetists to inspire, mentor and act as role models for future generations of anaesthetists.
- 2) There will be fewer high profile anaesthetists to inform medical students of the benefits of a career in anaesthetics, at the stage in their training when it is a serious consideration. This is particularly pertinent as recent studies show that medical students have significantly less interest in anaesthetics than other medical specialties [21].
- 3) The paucity of merit awards for anaesthetists overall means fewer awards will be available for IMGs. Raising the possibility that ambitious IMGs with an interest in anaesthetics will be less attracted to British healthcare.

Despite the relatively small number of anaesthetist merit award winners our data shows that the greatest proportion of IMGs, 33%, is represented in the tier 1 merit awards. Even taking into account the small numbers of awards involved, this may be an augury of what future IMGs may achieve, should the number of merit awards to anaesthetists be increased.

### Merit awards; undergraduate and postgraduate training of anaesthetists and non-anaesthetists

This research project is unique in investigating the relationship between national award-winning anaesthetists and their originating medical schools. Additionally and more specifically, little peer reviewed work has been published which investigates the effectiveness of each medical school in training their students and relates this to the future postgraduate success of each medical school's alumni. We were only able to identify three authoritative studies [21, 25]. The MedDifs study by McManus et al*.* [21] was the most authoritative and included some components that were comparable to our study. The MedDifs study involved examining UK medical school performances using 50 different criteria that were either quantitative or qualitative in nature. These criteria were grouped into categories [21]:

- Selection of applicants
- Student satisfaction
- Curricular influences
- Fitness to practise
- Choice of training specialty
- Postgraduate examination performance
- Foundation entry scores
- Perception of Foundation Year 1
- Teaching/learning and assessment
- Institutional history

In comparing our study to the MedDifs study, we were obviously more limited in the number of factors pertinent to medical education that we considered and we followed a purely quantitative approach to the research. Unsurprisingly, McManus et al*.* were able to correlate their range of factors and reveal educational relationships. For example:

- Medical schools that focused on Problem Based Learning tended to produce doctors that scored lower in postgraduate exams.
- Doctors from the bigger medical schools tended to score worse in postgraduate exams.
- Medical schools that focused on self-regulated learning produced doctors that tended to perform better in postgraduate exams.

Both their study and ours shared the limitation of not being able to assess and compare medical school courses in undergraduate medical degrees. Furthermore, the MedDifs project was much more limited in its ability to identify causal relationships between its investigated educational factors.

For the purpose of investigating possible causal relationships in our presented medical school rankings for anaesthetists, non-anaesthetists and all merit award-winners (Table 1), we examined the history of each of the UK university medical schools [26–35]. We were interested to find that all seven of the oldest university medical schools in the UK, calculated by their establishment dates, were represented in our top ten university medical school rankings by award-winners for anaesthetists, non-anaesthetists and aggregated total awards. These seven medical schools were formally established prior to 1826 and were Edinburgh (1726), St George's London University (1733), Glasgow (1751), St Bartholomew's university (1785), Aberdeen (1786), Manchester (1824) and Birmingham (1825) medical schools. Moreover, Oxford medical school was documented to have started teaching medicine in the twelfth century and Cambridge was known to have been teaching medicine from 1524; in essence, these two medical schools had been teaching clinical medicine before the formal establishment procedure had even been formed. Consequently, it becomes apparent that for each of our top 10 university medical school rankings (Table 1), *8 are descended from the oldest medical schools in the UK.*

Furthermore, considering medical schools established after 1999, none are present in the top 10 medical school rankings (Table 1). So, Warwick (2000), Norwich (2000), Peninsula (2000), Brighton and Sussex (2002), Hull York (2003), Keele (2003) and Swansea (2004) are not represented our top 10 (or top 20) medical school award-winner rankings. While it is understandable that medical schools established within the last ten years have not yet have had time for their graduates to distinguish themselves to national merit award levels, it is less clear that this explanation accounts for the absence of representation in the top 10 rankings, for medical schools established since the year 2000.

**Fig. 1:**
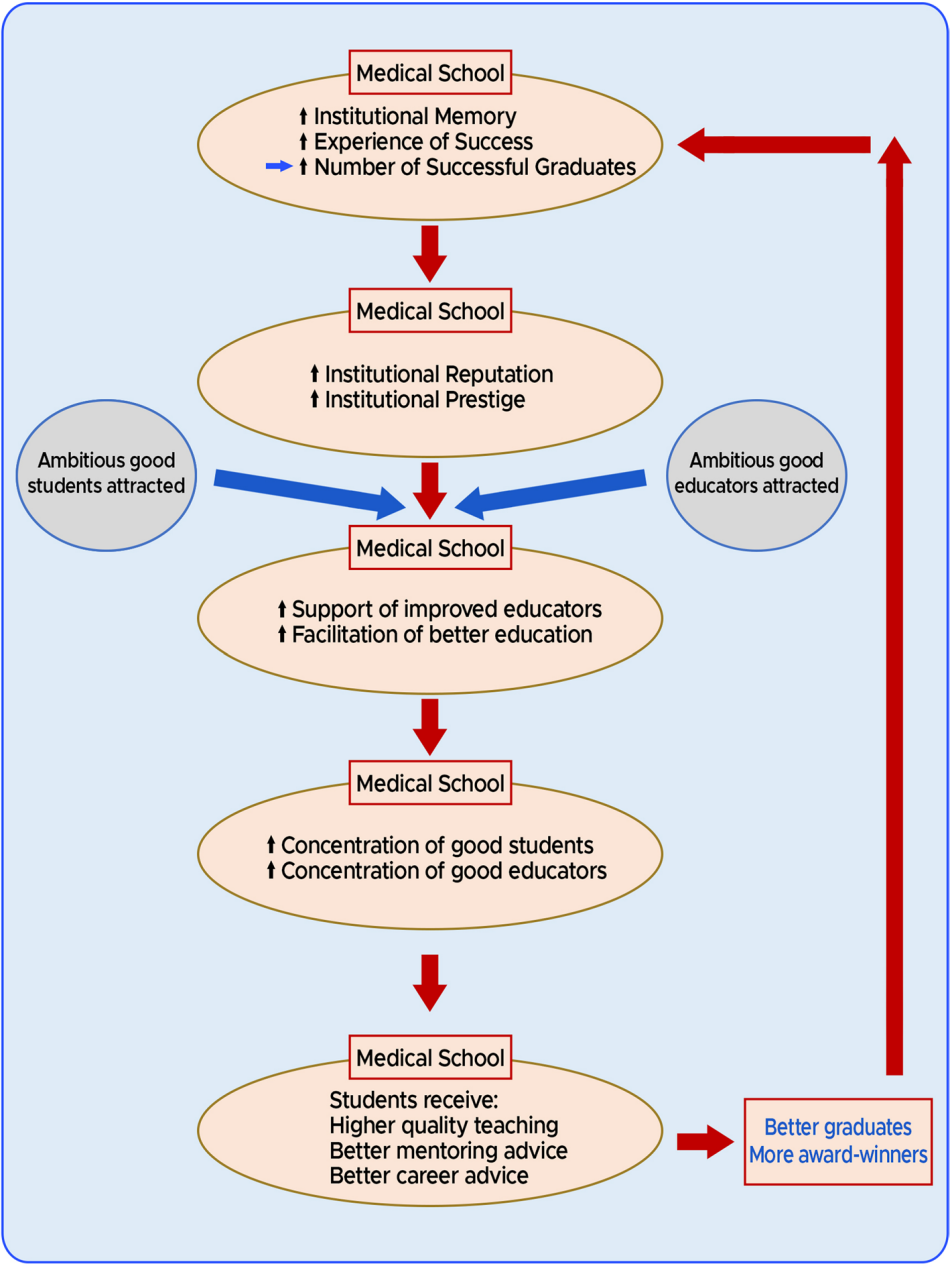
A model for the creation of award-winners. *Cycles of institutional memory and experience*.^16,17^

*In conclusion, our observations are consistent with at least a correlation between the age of the medical school and the number of subsequent graduates becoming merit award-winners.* In fact, we calculated a Pearson’s coefficient to determine the correlation between the age of the medical school by establishment date and the number of award winners corrected by size, for the top 20 medical schools, and found a significant correlation r(18) = 0.47, *p* = 0.04. After considering the totality of the results of our research study and also accepting the previous results of studies in UK medical school education [21, 25], in Fig. 1 we reiterate our model first described, elucidated and published earlier in this series [13, 14, 36, 37]. This model accounts for the age-dependent differential medical school performance in creating award-winning anaesthetists**:**

#### Cycles of Institutional Memory and Experience

- 1) Because of their greater age, the older university medical schools have accrued more institutional memory and experience in medical education than the more youthful medical schools. Accordingly, the older medical schools have a better chance of generating successful graduates—potentially before some of the younger medical schools have even become established.
- 2) As the older university medical schools appear to produce larger numbers of obviously successful alumni, they will garner positive reputations and inevitably be designated as more prestigious institutions. Consequently, ambitious, competent and career-focused students are more likely to be apply to these university medical schools.
- 3) Having produced more successful students, these older university medical schools will also have accumulated more experience in positively managing and educating such high-achieving students. Such experience will also coincide with improved support for the better educators in the medical school.
- 4) As a result, these older university medical schools with greater institutional memory and experience will tend to progressively and steadily accumulate a greater percentage of the most able students and educators.
- 5) Ultimately, the students in these older medical schools will tend to receive and benefit from better quality teaching, better mentoring and better medical career advice.
- 6) Thus, these older university medical schools will produce better educated, better advised and better prepared doctors who are more likely to become merit award-winners. There will also be an additional benefit to the originating medical school of having trained such high-achievers; they will accumulate greater experience in training award-winners, so adding to the institutional memory of successful education. *The cycle will then repeat.*

The medical education consequences of the action of *Cycles of Institutional Memory and Experience* can be described as follows:

- An inevitable result of the operation of the adjacent cycle is that the longer established medical schools have naturally experienced more cycling during their longer existences. This causes an accumulation of an increasing number of award-winners in the medical community, from each such originating medical school.
- The differential accumulation of award-winners in the community from each medical school depends on the relative efficacy and efficiency of the cycle in each medical school. Such efficiency differences account for the ultimate medical school rankings.
- The same considerations that led to development of the Cycles of Institutional Memory and Experience can also apply to the college/departmental/faculty levels. Specifically, a department that generates merit award-winning anaesthetists will tend to generate more such anaesthetists in the future. In principle, this could be termed a *departmental cycle of memory and experience*.

Any award scheme designed and administered by human beings runs the risk of introducing biases, thus leading to overrepresentation of particular groups. Our model provides a natural explanation and mechanism for connecting excellence/success with such bias. With every cycle of our model, increasing numbers of successful graduates originating from the older universities accumulate in the UK medical community. Subsequently, such distinguished and visible alumni are more likely to be elevated to senior leadership or managerial positions. These positions would include clinical excellence/distinction award allocators. Consequently, explicit selection biases or implicit selection biases would have a tendency to favour the graduates of these same medical schools of origin—resulting in a disproportionate number of these alumni gaining awards. Ultimately, we believe our model of *Cycles of Institutional Memory and Experience,* at least in part accounts for the concurrence of appropriate success/excellence in award-winning and any apparent bias in these medical school rankings. Consequently, it appears inevitable that the effects of genuine appropriate award achievement and bias are linked and would tend to be expressed simultaneously.

In the last 18 months there has been a reorganization of the UK national clinical excellence scheme. Specifically, in January 2022, it was announced that the latest iteration would be termed the "National Clinical Impact Awards, NCIA [38]." The governing authority announced that the objectives of this scheme would be to:

- Widen access.
- Simplify the application process, attempting to make it more equitable and inclusive.
- Reward excellence in a wider range of activities and behaviours [39].

This new rewards scheme offers a natural test and challenge to our *Cycles of Institutional Memory and Experience* model. Our model is based on the history and epidemiology of medical education in the UK. Accordingly, an analysis of the medical schools of origin of the NCIA winners should yield rankings similar to those reported in our series of publications, assuming that there is an underlying value to the model. We look forward to testing our model in this way.

## Conclusions

Our original study uses national clinical award-winning as an outcome measure to add training and education data to the demographic description of successful doctors in Britain. Specifically, we determine and present the university medical schools which are most likely to generate award-winning anaesthetists. We also determine and present university medical schools most likely to generate award-winning non-anaesthetists. *This study is the first to calculate and present a ranking of university medical schools by the number of national award-winning anaesthetists.* We provide a credible model to account for such rankings. Accordingly, we present comparative medical school data that can be used in the rational choice of medical schools for ambitious anaesthetist inclined, non-anaesthetist inclined and undecided medical school applicants.

We demonstrate that international medical graduates are making notable contributions to good anaesthesiological clinical practice in Britain, as judged by the number of award winners and their concentration amongst the tier 1 national merit award-winners. We provide evidence that indicates globalization and diversity of medical school origin are being reflected in the merit awards, indicating that Britain is a credible destination for ambitious medical trainees that seek national or international success.

## Data Availability

Data from this article is available upon reasonable request to the authors. Dr Sinclair Steele is the corresponding author and will make the data available. https://www.sehd.scot.nhs.uk/publications/DC20200319SACDA.pdf https://www.gov.uk/government/publications/accea-annual-report-2020 https://www.gmc-uk.org/registration-and-licensing/the-medical-register https://olr.gdc-uk.org/SearchRegister

## Abbreviations

IMG: International Medical Graduate
NCIA: National Clinical Impact Awards
CEA: Clinical Excellence Awards
DA: Distinction Awards
